# The substitution spectra of coronavirus genomes

**DOI:** 10.1093/bib/bbab382

**Published:** 2021-09-13

**Authors:** Diego Forni, Rachele Cagliani, Chiara Pontremoli, Mario Clerici, Manuela Sironi

**Affiliations:** Scientific Institute IRCCS E. MEDEA, Bioinformatics, Bosisio Parini, Italy; Scientific Institute IRCCS E. MEDEA, Bioinformatics, Bosisio Parini, Italy; Scientific Institute IRCCS E. MEDEA, Bioinformatics, Bosisio Parini, Italy; Department of Physiopathology and Transplantation, University of Milan, Milan, Italy; Don C. Gnocchi Foundation ONLUS, IRCCS, Milan, Italy; Scientific Institute IRCCS E. MEDEA, Bioinformatics, Bosisio Parini, Italy

**Keywords:** SARS-CoV-2, coronavirus, substitutions, transitions, transversions, RNA viruses

## Abstract

The severe acute respiratory syndrome coronavirus 2 (SARS-CoV-2) pandemic has triggered an unprecedented international effort to sequence complete viral genomes. We leveraged this wealth of information to characterize the substitution spectrum of SARS-CoV-2 and to compare it with those of other human and animal coronaviruses.

We show that, once nucleotide composition is taken into account, human and most animal coronaviruses display a mutation spectrum dominated by C to U and G to U substitutions, a feature that is not shared by other positive-sense RNA viruses. However, the proportions of C to U and G to U substitutions tend to decrease as divergence increases, suggesting that, whatever their origin, a proportion of these changes is subsequently eliminated by purifying selection. Analysis of the sequence context of C to U substitutions showed little evidence of apolipoprotein B mRNA editing catalytic polypeptide-like (APOBEC)-mediated editing and such contexts were similar for SARS-CoV-2 and Middle East respiratory syndrome coronavirus sampled from different hosts, despite different repertoires of APOBEC3 proteins in distinct species. Conversely, we found evidence that C to U and G to U changes affect CpG dinucleotides at a frequency higher than expected. Whereas this suggests ongoing selective reduction of CpGs, this effect alone cannot account for the substitution spectra. Finally, we show that, during the first months of SARS-CoV-2 pandemic spread, the frequency of both G to U and C to U substitutions increased. Our data suggest that the substitution spectrum of SARS-CoV-2 is determined by an interplay of factors, including intrinsic biases of the replication process, avoidance of CpG dinucleotides and other constraints exerted by the new host.

## Introduction

Severe acute respiratory syndrome coronavirus 2 (SARS-CoV-2), the causative agent of the ongoing COVID-19 pandemic, belongs to the *Sarbecovirus* genus in the *Coronaviridae* family. Coronaviruses are positive-sense RNA viruses that infect a large range of animal hosts. Up to now, at least seven coronaviruses have crossed the species barrier and spilled over to humans from a zoonotic source. Three are highly pathogenic (SARS-CoV-2, SARS-CoV and MERS-CoV), whereas the other four (HCoV-OC43, HCoV-NL63, HCoV-229E and HCoV-HKU1) are endemic in human populations and usually cause mild symptoms. The pathogenicity determinants of these viruses are still poorly understood but most likely impinge on multiple host processes [1–8].

It is still unknown whether the SARS-CoV-2 pandemic was initiated by the spillover from an intermediate host, but it is clear that the virus evolved in bats and that it required little adaptation to become a successful human pathogen [9, 10]. Since its introduction in the human host, though, SARS-CoV-2 most likely experienced different selective pressures than in the bat reservoir. Of course, these include the human innate and adaptive immune systems.

Because they encode enzymes with proofreading ability [11–13], coronaviruses typically have lower mutation rates compared with other RNA viruses [14]. Indeed, the estimated substitution rate of SARS-CoV-2 is around 10^−3^ substitutions per site per year [15–17], meaning that circulating viruses accumulate ~2 substitutions per month. This is in line with the observation that, during the first 6 months of the pandemic, viral genetic diversity remained limited. However, since September 2020, highly divergent viral lineages appeared in different geographic locations [18]. Such lineages are characterized by a remarkable number of sequence changes, and some of them have been designated as variants of concern (VOCs; e.g. B.1.1.7, B.1.351 and P.1; [18]). The mechanisms underlying the emergence of these new viral lineages are presently unknown [19–21].

The mutation spectrum of SARS-CoV-2 has been investigated in several studies, with a general consensus that it is dominated by C to U substitutions [22–30]. However, G to U changes also occur at unusually high rates [22, 24, 26, 31]. This was proposed to be due to the effect of reactive oxygen species (ROS), which can cause guanine oxidation to 8-oxo-7,8-dihydroguanine (8-oxoguanine). 8-oxoguanine can pair with adenine, ultimately causing G to U transversions [24, 28, 31]. Conversely, the excess of C to U changes was proposed to be consistent with the action of APOBEC (apolipoprotein B mRNA editing catalytic polypeptide-like) proteins, which function as cellular cytosine deaminases [22–26, 28, 30].

Placental mammals display a variable number of APOBEC3 (A3) paralogs, many of which act as antiviral effectors. Although the specific nature of their substrates is still to be clarified, the seven A3 proteins encoded by humans (and great apes) function as deaminases and display RNA binding activity. So far, however, the ability to deaminate RNA has only been demonstrated for A3A and A3G (rev in [32]). A3 proteins are known to differ in their context preferences: A3A, A3B, A3C, A3D, A3F and A3H primarily target 5′-TC-3′ motifs, whereas A3G targets 5′-CC-3′ [32]. Other mammals display very different repertoires of A3 proteins, which are absent in nonmammalian vertebrates [32]. An additional member of the APOBEC family, APOBEC1 has the major function of editing the 3′UTRs of cellular transcripts and typically targets AU-rich sequences. APOBEC1 originated by duplication of AID before the tetrapod-lungfish divergence [33]. However, no APOBEC1 gene is present in the genome of chicken, possibly as a result of secondary loss [34]. These differences in the repertoires of APOBEC proteins suggest that comparison of the mutation pattern of SARS-CoV-2 with those of coronaviruses that infect different mammalian and nonmammalian hosts can provide information about whether and which cellular processes drive the substitution pattern of these viruses.

Of course, APOBECs are not the only cellular proteins that counteract viral infections. For instance, the zinc-finger antiviral protein (ZAP) is induced by interferons and can restrict several viruses, including SARS-CoV-2 [35]. ZAP specifically binds CpG dinucleotides in single-stranded RNA [36] and recruits the RNA processing exosome to degrade its targets [37]. The antiviral activity of ZAP is thought to drive the depletion of CpG dinucleotides observed in the genomes of several viruses that infect mammals, including human coronaviruses [10, 35, 38, 39]. In particular, analysis of sarbecoviruses indicated that a strong depletion of CpG dinucleotides in SARS-CoV-2 and other closely related viruses occurred during evolution in bats [10].

Here, we exploit the enormous amount of available SARS-CoV-2 genomes, as well as sequence data for other coronaviruses and positive-strand RNA viruses to study substitution spectra. Whereas our results provide little evidence for the previously suggested roles of ROS or APOBEC in shaping substitution frequencies [22–26, 28, 30, 31], they show that depletion of CpG dinucleotides is ongoing in SARS-CoV-2 and other viruses. Also, analysis of substitution spectra over the first year of SARS-CoV-2 pandemic spread indicated a change in substitution frequencies in the initial months, possibly as a result of new selective pressures ensuing from the host shift.

## Materials and methods

### Sequence collection

SARS-CoV-2 sequences were retrieved from the global initiative on sharing avian influenza data (GISAID) Initiative database (as of 13th April 2021, https://www.gisaid.org). A Multiple Alignment using Fast Fourier Transform (MAFFT)-generated alignment of high coverage complete genome sequences was downloaded from the website. Only strains derived from human hosts were selected, generating a set of 804 779 sequences. A set of SARS-CoV-2 genomes sequenced from minks sampled in different geographic locations was also retrieved from the GISAD database.

We also analyzed the substitution patterns of different coronaviruses: SARS-CoV, Middle East respiratory syndrome coronavirus (MERS-CoV), human coronavirus OC43 (HCoV-OC43), human coronavirus NL63 (HCoV-NL63), bovine coronavirus (BCoV), porcine deltacoronavirus (PDCoV), porcine epidemic diarrhea virus (PEDV), feline coronavirus (FCoV) and avian coronavirus infectious bronchitis virus (IBV). For all these coronaviruses, as well as for a set of positive-strand RNA viruses, complete genome sequences were retrieved from the National Center for Biotechnology Information database (NCBI, http://www.ncbi.nlm.nih.gov/). Only coronaviruses with an adequate number of complete available genomes were included in the analyses. All NCBI viral sequence IDs are listed in Supplementary Table 1. All genome alignments were generated using the MAFFT software (v7.427) [40], with default parameters.

### Estimation of substitution pattern

Substitution patterns were calculated by comparing all positions of each viral genome alignment with a reference sequence. For each viral species, the sequence with the earliest collection date was used as the reference (Supplementary Table 1). To partially account for the phylogenetic relationship among genomes and for sequencing errors, for each position we counted once all identical mismatches that occurred in at least two sequences. By using this approach, we did not consider substitutions that revert to the reference alleles, but we counted substitutions when different nucleotides were present at the same position.

Proportions of substitutions (relative to the overall number of mismatches for a given virus genome alignment) were then normalized by the total number of occurrences of the nucleotide in the reference genome (e.g. G to U changes were normalized by the number of G bases that are present in the reference genome—i.e. *n*_GtoU_/(*n*_G*_n_overall_substitutions_)).

In the case of SARS-CoV-2, we created 100 independent sets of 1000 randomly selected sequences (i.e. from the total set of 804 779 genomes, 100 000 sequences were randomly sampled and divided into 100 sets).

Virus pairwise divergence was calculated using the ape R package [41].

### Analysis of mismatch sequence context

The sequence contexts (−1 and +1 nucleotide positions) were analyzed for the two most skewed substitutions. Again, because dinucleotide composition is biased in most viral genomes [42–44], we normalized counts by the frequency of the specific dinucleotide involved in the mismatch. In particular, counts of the 4 nucleotides flanking C to U and G to U mismatches were divided by the overall number of 5′-NS-3′ or 5′-SN-3′ in the reference genome (where N is one of the four bases and S is either C or G). These frequencies were then normalized by the overall counts of each specific mismatch.

For SARS-CoV-2, C to U and G to U sequence intervals were also evaluated in the context of coding sequence frame (based on the reference sequence genome annotation). Mismatches were included only if they occurred at the third codon position and only synonymous changes were considered.

Thus, for C to U substitutions, we counted the number of C to U occurring in third codon positions (i.e. NNC to NNU) and we counted which nucleotide was at the first position for the next codon. These counts were then normalized by dividing for the overall number of dinucleotides composed by C at third codon positions and for each of all four nucleotides at the first position of the next codon in all ORFs.

In the case of G to U changes, we retrieved codons having this substitution at the third position (i.e. NNG to NNU) and we counted which nucleotide was present in the second position of the same codon. No codon with an A in the second position allows a synonymous G to U substitution in the third position. However, such synonymous substitutions are allowed for the other three nucleotides, with C having four synonymous codons and U and G, having two. We thus counted the frequency of these codons with a G to U substitution by normalizing for their overall counts. A similar approach was used to analyze the frequency of C to U synonymous substitutions at 4-fold/3-fold-degenerate codons that contain or do not contain an UpC dinucleotide.

### Substitution pattern over time

For SARS-CoV-2, C to U and G to U mismatches were analyzed in time intervals. Substitution patterns were calculated as described above, but in this case sequences were grouped based on collection date (by months) and only new mismatches were considered (i.e. mutations already present in previous months were discarded). Because countries where VOCs are detected tend to intensify genomic surveillance and because VOCs may be preferentially sequenced, we removed VOC lineages from these analyses to avoid sampling biases. To evaluate the effect of sampling and to create comparable sets among months, we created for each month 10 independent sets of 500 randomly selected sequences. This was not possible for January and February 2020, because these time points included 497 and 500 sequences, respectively. For these two intervals, all sequences were analyzed and the number of the sequences in January and February set the choice of 500 sequences/month.

## Results

### Analysis of substitution patterns

**Figure 1:**
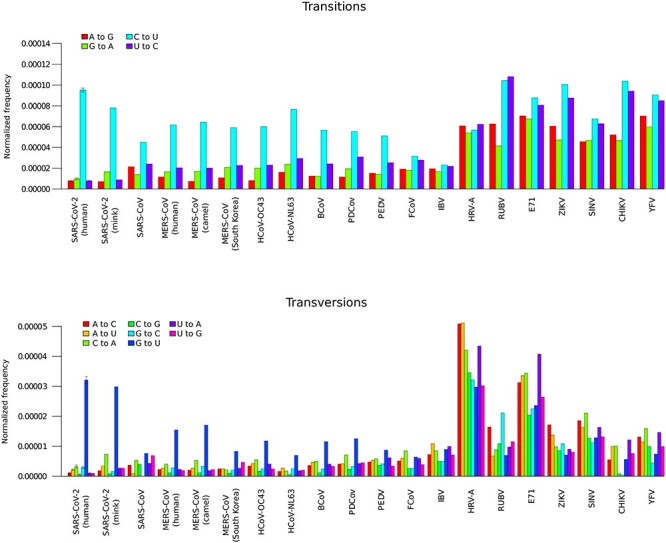
Transition and transversion frequencies in coronaviruses and other positive-sense RNA viruses. Transition and transversion frequencies are reported after normalization by base frequency and by overall number of mutations. Data for SARS-CoV-2 are plotted as mean and standard deviation of 100 sets of 1000 genomes each. For all other viruses, the sequences analyzed were as follows: mink SARS-CoV-2 = 880, SARS-CoV = 48, human MERS-CoV = 314, camel MERS-CoV = 342, South Koean MERS-CoV = 34, HCoV-OC43 = 167, HCoV-NL63 = 68, BCoV = 84, PDCoV = 124, PEDV = 471, FCoV = 52, IBV = 404, HRV-A = 198, E71 = 994, RUBV = 82, ZIKV = 659, SINV = 76, CHIKV = 157 and YFV = 35.

We first aimed to characterize the substitution patterns of SARS-CoV-2 and other coronaviruses, as well to compare them with those of other positive-sense RNA viruses that infect humans. Given the enormous amount of sequence data, for SARS-CoV-2, we generated 100 sets of 1000 sequences each. These were selected to be complete genome sequences with high coverage (see Material and Methods). For each set, we counted the number of different transitions and transversions with respect to the Wuhan-Hu-1 reference sequence. Average values and standard deviations were calculated to estimate the variability of mutation frequencies. To partially account for the nonindependence among genomes, we counted once all mismatches that occurred in at least two sequences (i.e. if the same change occurred in more than two sequences it was considered to have appeared in an ancestor). Because the probability that a specific nucleotide mutates also depends on its frequency in the genome, counts were normalized by the number of each nucleotide (e.g. G to U changes were normalized by the number of G bases) and by the overall number of mutations. As previously observed [22–30], the mutation spectrum of transitions was dominated by C to U substitutions, whereas G to U was, by large, the most common transversion (Figure 1). As expected, the frequency of all substitutions was higher in accessory and structural proteins than in the nonstructural ones (Supplementary Figure 1).

To compare the substitution pattern with that observed during the evolution of SARS-CoV-2 in a nonhuman host, we analyzed 880 viral genomes sequenced from minks, which were infected during large spillovers in different locations [45]. Again, a strong asymmetry towards G to U and C to U substitutions was observed (Figure 1).

Previous studies showed that other human coronaviruses also display elevated ratios of C to U and G to U substitutions [23, 30, 31]. We thus analyzed the substitution spectra of SARS-CoV, HCoV-NL63, HCoV-OC43, as well as of MERS-CoV. In this latter case, sequences were separated based on the host they were sampled from (humans or camels). It is however worth mentioning that, because MERS human cases resulted from multiple spillovers, it is possible that a proportion of the observed substitutions in the human-derived sequences were inherited from the diversity within camels (rather than being changes that occurred in humans; [46]). To check for this possibility, we separately analyzed 34 MERS-CoV genomes that were sampled during the 2015 outbreak in South Korea, which was caused by a single introduction by a traveler returning from the Middle East [47]. We also analyzed the substitution patterns of coronaviruses that infect other animals (cattle, pigs, cats and chickens). These viruses, as well as the human ones, were selected on the basis of the number of available sequences in public repositories.

Results indicated that all coronaviruses, with the exclusion of IBV (avian coronavirus infectious bronchitis virus) and FCoV (feline coronavirus), have a transition frequency skewed towards C to U changes (Figure 1). Likewise, with the exception of IBV and FCoV, G to U substitutions were the most common transversions in all coronaviruses. Notably, SARS-CoV, which is the human virus closest to SARS-CoV-2, displayed the lowest preference for G to U transversions among coronaviruses (Figure 1). To check whether these differences might be due to the number of available complete genomes, which is clearly much lower for SARS-CoV (*n* = 48) than for SARS-CoV-2, we applied a resampling approach. Thus, 48 SARS-CoV-2 sequences were randomly selected 10 times and analyzed. Little variation was observed and the frequency of G to U changes was always much higher for SARS-CoV-2 than for SARS-CoV (Supplementary Figure 2).

With respect to MERS-CoV, the substitution pattern was similar in the full set of human-derived sequences and in the ones sampled in South Korea, although the latter showed a less marked preference for G to U changes (Figure 1). Overall, this suggests that the similarity of the substitution patterns in the human and camel host is not due to the fact that most changes are inherited from camels.

We next wished to determine whether the same mutation pattern was shared with other positive-sense RNA viruses. We thus analyzed sequence data for Zika virus (ZIKV, family *Flaviviridae*), Sindbis virus (SINV, family *Togaviridae*), human rhinovirus A (HRV-A, family *Picornaviridae)*, enterovirus E71 (family *Picornaviridae*) and rubella virus (RUBV, family *Metonaviridae)*. To have a glimpse of substitution patterns over short time frames, we also inspected 156 Chikungunya virus (CHIKV, *Togaviridae*) sequences from the 2013 to 2017 South American epidemic (Asian Urban American genotype; [48]), as well as 34 Yellow fever virus (YFV, *Flaviviridae*) genomes from the 2016 to 2017 epidemic in Brazil [49]. None of these viruses showed a mutation pattern similar to that of coronaviruses and there was no clear over-representation of C to U or G to U changes compared with other transitions/transversions (Figure 1).

**Figure 2:**
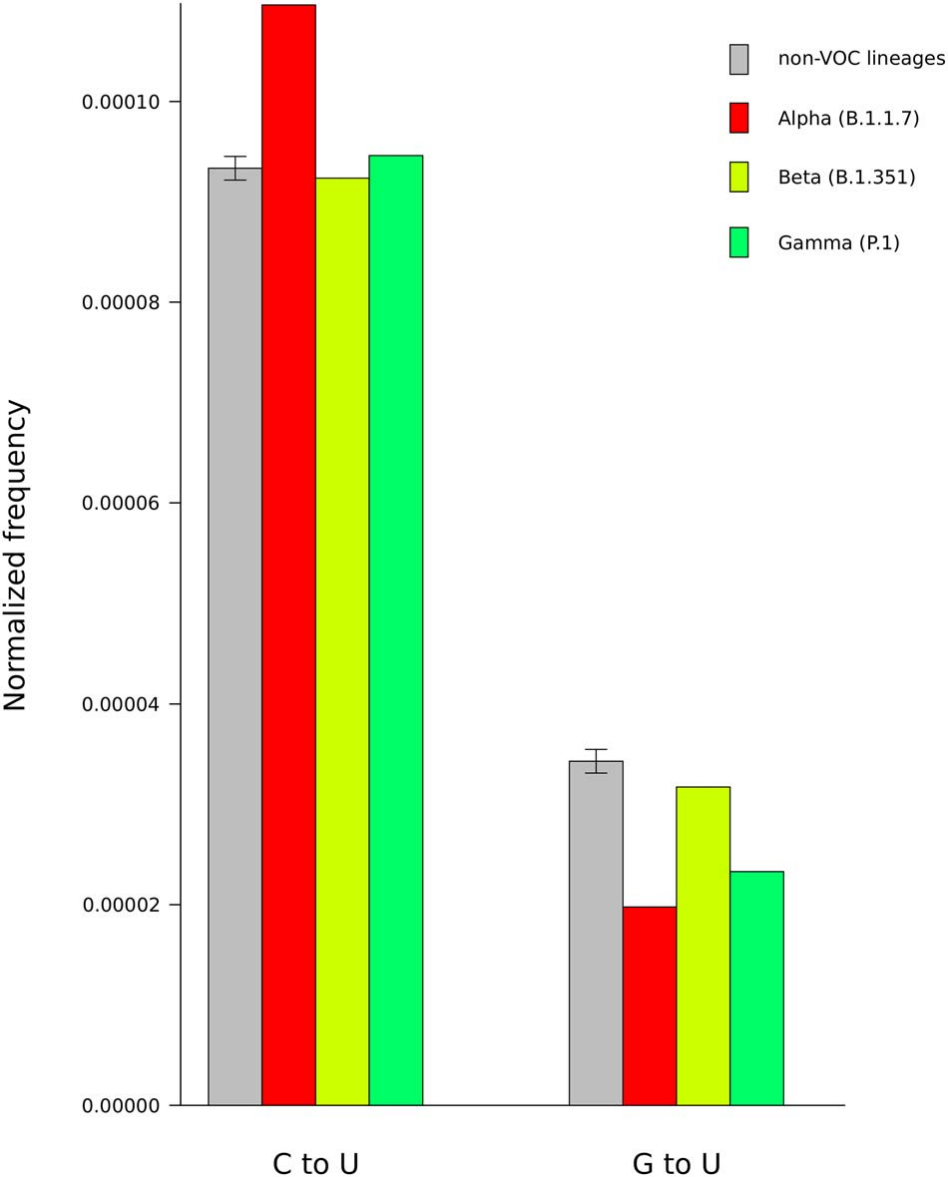
C to U and G to U substitutions frequencies for VOCs and non-VOC lineages. Substitution frequencies are reported after normalization by base frequency and by overall number of mutations. Data for non-VOC genomes are plotted as mean and standard deviation of 100 sets of 1000 genomes each.

Finally, we compared SARS-CoV-2 VOC with non-VOC sequences and we found no major differences in C to U and G to U substitution frequencies (Figure 2).

Substitutions are generated by mutation or other processes (e.g. RNA editing). Over time, however, the fate of sequence changes is affected by natural selection, genetic drift, founder effects and recombination. Consequently, substitutions observed over different times frames differently reflect the relevance of mutation versus that of other processes. To account for this, for all viruses analyzed above, we calculated the average pairwise divergence and we compared it with the proportion of C to U and G to U changes (over all other transitions or transversions).

**Figure 3:**
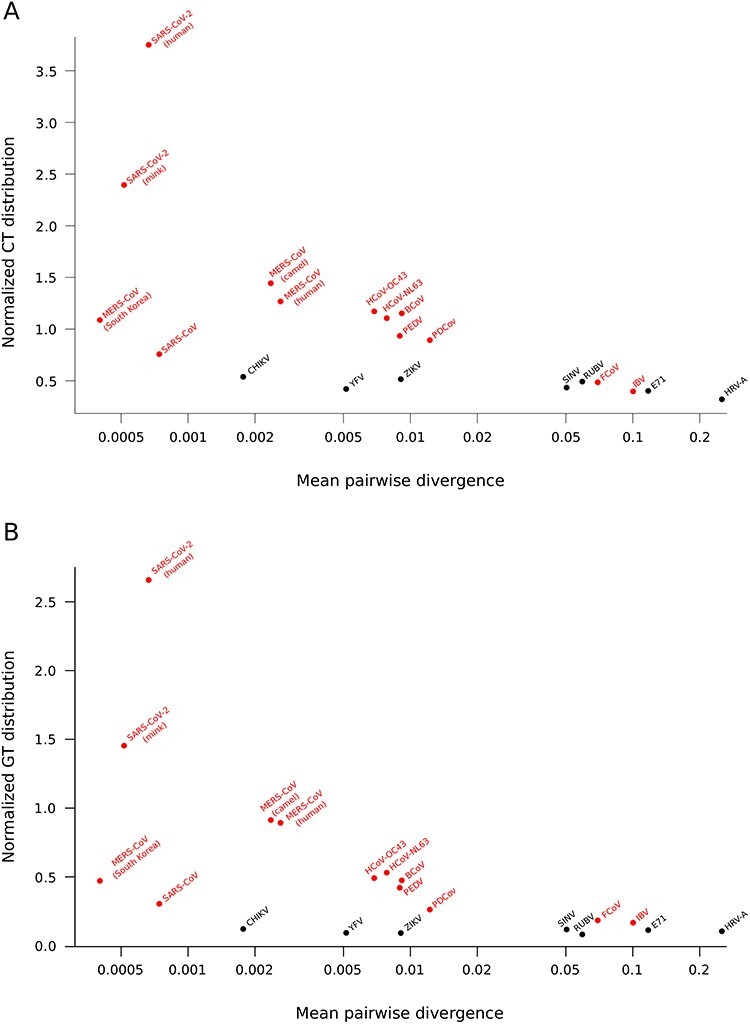
Correlation between substitutions and sequences divergence. C to U and G to U substitution frequencies are plotted against the mean pairwise divergence for coronaviruses (red dots) and a set of positive strand RNA viruses (black dots). C to U and G to U frequencies are calculated as described in Figure 1 and then divided by the sum of the other transition and transversion substitutions, respectively.

For coronaviruses, results (Figure 3) indicated that the proportions of C to U and G to U changes tend to decrease as divergence increases. However, the trends were mainly driven by SARS-CoV-2 on one end and by IBV and FCoV on the other. For instance, SARS-CoV sequences displayed low divergence and low proportions of C to U and G to U substitutions, as also evident in Figure 1. Conversely, no change of substitution proportions with divergence was evident for other positive-sense RNA viruses, as recently diverged sequences also showed fewer C to U and G to U changes than coronaviruses. It is however worth mentioning that ZIKV, CHIKV and YFV are mosquito-borne viruses and therefore their substitutions spectrum results from mutation/selection processes that occur in both the vertebrate host and in the invertebrate vector.

### Sequence context of substitutions

Analysis of the sequence context of specific substitutions might provide clues about the mechanism(s) responsible for their generation. Previous studies indicated that, in the genomes of SARS-CoV-2 and of other human coronaviruses, C to U substitutions preferentially occur in AU-rich sequence contexts [23, 30]. However, coronaviruses tend to have AU-rich genomes (e.g. the SARS-CoV-2 reference strain has a CG content of 37.99%) and the likelihood that a substitution falls within a given context is thus skewed. Moreover, as anticipated above, SARS-CoV-2 evolution was accompanied by a depletion of CpG dinucleotides, most likely to evade antiviral cellular systems [10]. We thus analyzed the sequence context (−1 and +1 positions) of C to U and G to U changes by taking dinucleotide composition into account. Thus, the frequency of C to U substitutions was normalized by the number of 5′-NC-3′ and 5′-CN-3′ dinucleotides. The same approach was used for G to U changes. Because the overall number of substitutions was limited, SARS-CoV, YFV and the South Korean MERS-CoV samples were not analyzed.

**Figure 4:**
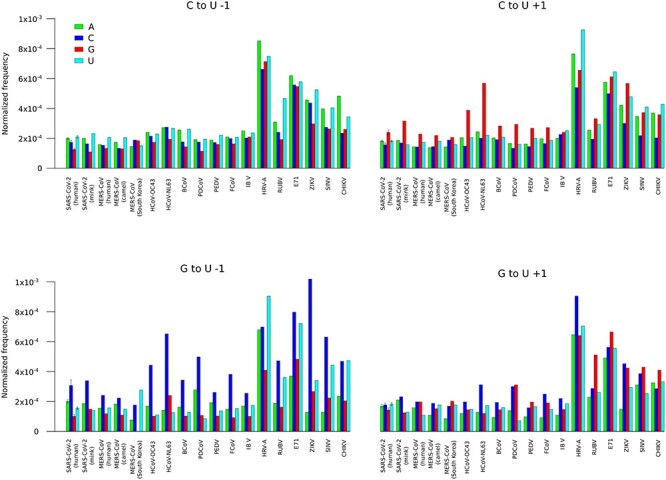
Sequence context of C to U and G to U substitutions in coronaviruses and other positive-sense RNA viruses. The frequency of nucleotides flanking (−1 and +1 positions) C to U and G to U changes are reported after normalization by dinucleotide genome composition. Data for SARS-CoV-2 are plotted as mean and standard deviation of 100 sets of 1000 genomes each.

For most coronaviruses, we found a marked preference for guanosines in position +1 of C to U changes and for cytosines in position −1 for G to U substitutions. This latter effect was also evident for other RNA viruses and most notably for ZIKV and SINV (Figure 4). With the exclusion of RUBV, there was no marked over-representation of U nucleotides at the −1 position of C to U changes (A3A, A3B, A3C, A3D, A3F and A3H preference) or of cytosines either in +1 or −1 (A3G preference; Figure 4). Also, with the exclusion of IBV, the contexts were quite similar across coronaviruses irrespective of their hosts, which carry different repertoires of APOBEC3 proteins with distinct editing preferences (Figure 4; [32–34, 50]). However, some over-representation of A/U nucleotides was observed upstream C to U changes, possibly suggesting the action of APOBEC1 ([50]; Figure 4). Overall, these data indicate that a higher than expected fraction of C to U and G to U substitutions in coronaviruses targets CpG dinucleotides.

**Figure 5:**
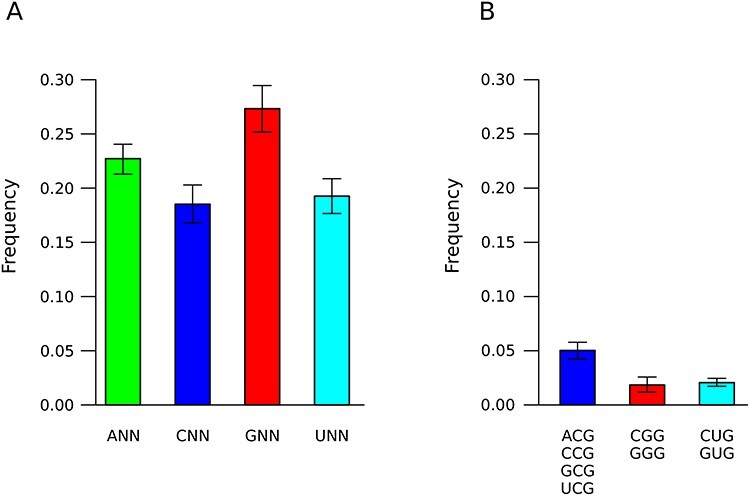
Sequence context of synonymous substitutions in SARS-CoV-2. (**A**) For C to U synonymous changes, bars represent the frequency of substitutions that occur at NNC codons when the next codon is GNN or HNN (where H is A or C or U). (**B**) For G to U synonymous changes, bars indicate the frequency of substitutions that change an NCG codon and those that change an NDG codon (where D is G or A or U). All data are plotted as mean and standard deviation of 100 sets of 1000 genomes each.

As is the case of most viruses, the majority of the coronavirus genome is protein-coding. As a consequence, substitutions have very different effects depending on whether they are synonymous or nonsynonymous. We thus assessed if the effect on CpGs was also observed when only synonymous changes were analyzed in the large SARS-CoV-2 dataset. Thus, for C to U changes, we calculated the frequency of synonymous substitutions that occur at NNC codons when the next codon is GNN or HNN (where H is A or C or U). The frequency was higher for NNC–GNN di-codons than for NNC–HNN ones (Figure 5). In the case of G to U changes, we compared the frequency of synonymous substitutions that change an NCG codon (containing a CpG) with those that change an NDG codon (where D is G or A or U, not containing a CpG). The frequency of changes at NCG codons was, on average, much higher than at NDG codons (Figure 5). A similar analysis of C to U changes at NUC codons (carrying the UpC preferred motif for may APOBECs) compared with NVC codons (where V is A or C or G) revealed no strong difference in frequency, confirming a limited role for A3A, A3B, A3C, A3D, A3F and A3H (Supplementary Figure 3).

Overall, these data suggest ongoing selection on CpGs in the genome of SARS-CoV-2 and, possibly, of other coronaviruses. However, again using the SARS-CoV-2 data, we estimated that a relatively minor proportion of C to U (10.34 ± 0.75%) and G to U (13.50 ± 1.29%) substitutions occur at CpG dinucleotides, which thus do not completely explain the skewed substitution pattern.

### Substitution spectra in time

**Figure 6:**
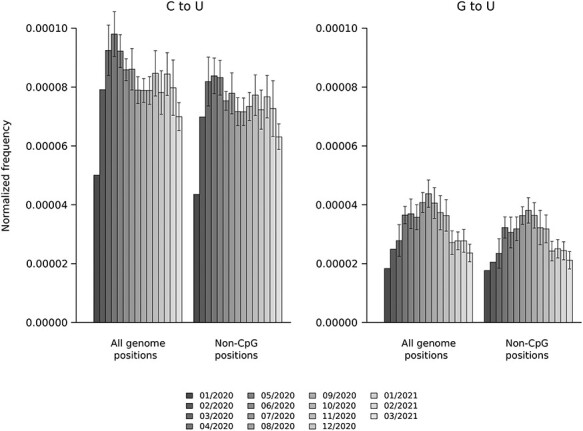
Change of the substitution spectrum over time. Frequency of C to U and G to U mutations that have appeared per month (see Materials and Methods). Frequencies are normalized by the frequency of the changing nucleotide and by the overall number of changes occurring each month. From March 2020 onward, data are plotted as mean and standard deviation of 10 sets of 500 genomes each. For January and February 2020, data represent frequencies calculated over 497 and 500 sequences, respectively.

Most studies that analyzed the SARS-CoV-2 mutation spectrum used sequences from the early phases of the pandemic [23, 25–27, 30], and possible changes in the substitution pattern with time have not been investigated. If present, these might ensue from shifts in the mutation or selection processes during the pandemic. We thus estimated the frequency of C to U and G to U mutations that are new to each month (i.e. mutations already present in earlier time points were not recorded) from January 2020 to March 2021. For each month, we sampled 500 sequences in ten sets. This was not possible for January and February 2020, and these time points included 497 and 500 sequences, respectively. As above, substitution proportions were normalized by the frequency of the changing nucleotide. For both C to U and G to U substitutions, results showed a steep increase in the first four months, a stabilization around April 2020, albeit with fluctuations and a decrease (Figure 6). A similar pattern was observed when we only analyzed substitutions that occur at non-CpG sites (Figure 6).

It is now established that, starting from March 2020, the B.1 SARS-CoV-2 lineage emerged and rapidly increased in frequency worldwide. This lineage is characterized by two nonsynonymous substitutions, the D614G variant in the spike protein and the P323L change in the viral RNA polymerase (RdRp; [51]). The P323L change was previously suggested to affect SARS-CoV-2 substitution rates [52] and mutations in the RdRp of other RNA viruses have been associated with changes in the mutation spectrum [53–56]. We thus compared the occurrence of C to U and G to U substitutions in genomes carrying P323 or L323. In particular, we analyzed the first six months of 2020 and the time period from December 2020 to March 2021 (in the second half of 2020 all sequences carry L323). Results indicated that the increase in C to U and G to U rates in the early phases of the epidemic occurred for both the P323 and the L323 lineages, which did not consistently differ in substitution frequencies across time (Supplementary Figure 4).

## Discussion

We have characterized the substitution spectrum of SARS-CoV-2 using >800 000 full-length, high-quality genomes. We confirm that the SARS-CoV-2 substitution pattern is highly skewed and dominated by C to U and G to U changes [22–27, 29–31]. More generally, our data indicate that, once the nucleotide composition of their genomes is taken into account, human epidemic and endemic coronaviruses, as well as most animal coronaviruses, display a substitution spectrum dominated by C to U and G to U changes, a feature which is not shared by other positive-sense RNA viruses. In fact, some of these latter show a marked preference for C to U (and U to C) changes, but not for G to U substitutions, as previously reported for RUBV [25].

Although the overall number of analyzed viruses does not allow to draw a firm conclusion, the C to U and G to U preference seems to be stronger for coronaviruses showing limited divergence. This suggests that, whatever their origin, a proportion of these changes is subsequently eliminated by purifying selection. Indeed, a large fraction of polymorphic variation in RNA virus populations is accounted for by transient deleterious mutations that are gradually purged by selection [57]. Thus, recently emerged viruses are expected to display a substitution pattern that reflects the mutation process more closely than those of viruses that have been subject to natural selection for longer time frames. Moreover, in the case of SARS-CoV-2, the exponential growth of the viral population [10] is likely to have reduced the efficacy of purifying selection and to have increased the proportion of segregating deleterious mutations [58]. These considerations might help explain why, among coronaviruses, SARS-CoV-2 displays the strongest preference for C to U and G to U changes, whereas FCoV and IBV, which have diverged more than other coronaviruses, have the weakest.

Analysis of substitution frequencies also indicated that the pattern is remarkably similar between sequences of the same virus sampled from different hosts (i.e. SARS-CoV-2 from humans and minks or MERS-CoV from humans and camels), whereas it can differ among coronaviruses that infect the same host. The situation is however complicated by the fact that distinct viruses may display different tissue tropism. For instance, human coronaviruses primarily infect the respiratory tract, whereas the bovine and porcine coronaviruses we analyzed herein are mainly enteric pathogens [59–61]. Nevertheless, the difference in G to U substitutions is striking between SARS-CoV-2 and SARS-CoV, which have similar (albeit not identical) tropism [62]. The difference between the two viruses is unlikely the result of limited sampling of the latter, either. Overall, these data suggest that substitution patterns tend to be more virus-specific than host-specific, questioning the idea that cellular antiviral mechanisms have a major role in shaping coronavirus substitution spectra [23–26, 28, 30]. Indeed, analysis of the sequence context of C to U changes showed little evidence of APOBEC3-mediated editing. With the exclusion of RUBV, which was previously suggested to be edited by APOBECs [63], we detected no strong and specific over-representation of uridines or cytidines flanking C to U changes. Also, the context of C to U changes was similar for SARS-CoV-2 sampled from humans and minks, as well as for MERS-CoV from humans and camels, although carnivores and artiodactyla have a very different complement of A3 proteins compared to humans, most likely with distinct target preferences [64]. Moreover, *in vitro* analyses showed that HCoV-NL63, which displays high frequencies of C to U changes, can be restricted, but not edited, by A3C, A3F and A3H [65]. Whereas it is possible that *in vitro* experiments do not recapitulate the situation *in vivo*, this observation adds evidence against the possibility that APOBEC3 proteins are major determinants of coronavirus substitution patterns.

With respect to APOBEC1, the protein is encoded by most vertebrates and its editing preferences for AU-rich sequences are conserved in humans and rodents [66–69]. At least for some coronaviruses and for SINV, we found a slight over-representation of A/U nucleotides, especially in the −1 position of C to U changes. However, the A/U over-representation is modest, indicating that even if APOBEC1 is implicated, its contribution to the overall substitution pattern is limited. Moreover, in analogy to observations on human Influenza A viruses, the A/U over-representation might also derive from the preferential depletion of CpG dinucleotides in AU-rich contexts [70] (see below). Overall, these data concur with previous observations [22] to indicate that the bulk of C to U changes in coronavirus genomes is not the result of cellular editing.

Whereas we found little evidence that the cellular APOBEC system plays a role in shaping the evolution of coronavirus genomes, we found that C to U and G to U changes affect CpG dinucleotides at a frequency higher than expected. This suggests ongoing selective reduction of CpGs, possibly to escape ZAP-mediated restriction. Such an observation is in line with data indicating that, despite its low CpG content [10, 35, 38, 39, 71, 72], SARS-CoV-2 can be restricted by ZAP [35]. In fact, MacLean *et al.* showed that adaptive depletion of CpG dinucleotiodes occurred on the lineage leading to SARS-CoV-2 during evolution in the bat reservoir. Whether this reduction in CpGs during the early evolution of SARS-CoV-2 was secondary to a change in tissue tropism or to adaptation to a different bat host is presently unknown [10]. Whatever the underlying reason, the residual sensitivity to antiviral cellular systems in humans most likely represents the selective force responsible for driving CpG depletion. Indeed, selective reduction in CpG frequencies has previously been noticed upon zoonotic transmission of influenza A viruses from birds to mammals and most likely reflects a different strength of selective pressure against CpG dinucleotides in different hosts [43, 73]. It is nonetheless interesting to notice that, based on the sequence context of C to U and G to U changes, selection against CpGs seems to be also ongoing for endemic human coronaviruses, animal coronaviruses, as well as other positive-sense RNA viruses such as ZIKV and SINV, these latter sensitive to ZAP [74, 75]. This is reminiscent of observations on influenza A viruses, which showed selection against CpG dinucleotides to act over decades [43, 73]. Overall, these data suggest that SARS-CoV-2 and other coronaviruses have been adapting to reduce their CpG content and that the equilibrium frequency has not been reached yet. However, we cannot formally exclude that substitutions at CpG sites are due to mutational biases rather than selection. In any case, the overall number of changes at CpGs is small and cannot account for the observed substitution spectra. Thus, the substitution patterns of coronaviruses remain largely unexplained.

Concerning G to U changes, previous studies [24, 28, 31] suggested a possible role of ROSs. Whereas we cannot rule out their contribution, it seems unlikely that ROSs are mutagenic for SARS-CoV-2 and other human coronaviruses, but have little effect on SARS-CoV. Also, in case ROSs were responsible for the bulk of G to U changes, these would not be expected to change in frequency with time. One possibility is that substitution patterns partially result from intrinsic mutational biases of the RdRp or other processes related to viral replication. In this case, though, symmetry in the substitution pattern would be expected, as coronaviruses replicate through negative-strand intermediates [62]. Although the underlying mechanisms remain to be clarified, amino acid substitutions in viral polymerases have previously been associated with both changes in fidelity and introduction of specific mutational biases [53–56]. These lines of evidence, together with the timing of C to U and G to U frequency increase, led us to hypothesize that the P323L change in the RdRp might contribute to mutation biases, as previously suggested [52]. Although we did not find evidence that this is the case, the possibility still exists that the observed patterns of substitution are in part accounted for by biases intrinsic to the molecular mechanism of coronavirus RdRp polymerase fidelity and/or proofreading ability of nsp14/nsp12. Concerning the latter, *in vitro* experiments with SARS-CoV indicated that the exoribonuclease activity does not depend on the mismatched base pair [76]. Thus, the increase in frequency of C to U and G to U changes, irrespective of RdRp mutations, during the first months of human infection also suggest some changes in mutation patterns and/or selective pressures since the spillover. This is in line with a recent study that analyzed differences between SARS-CoV-2 and closely related bat viruses [31]. The authors reached the conclusion that the G to U substitution frequency increased 9-fold after the introduction of SARS-CoV-2 in humans.

Understanding the mechanisms underlying the substitution pattern of SARS-CoV-2 is essential to appreciate its possible evolutionary paths in the long-term. The emergence of VOCs and their origin are still poorly understood events. Our data indicate that VOCs have a substitution spectrum very similar to that of other lineages. This is in line with the observation that B.1.1.7 and P.1 evolve at a similar rate as non-VOC strains [20, 21]. Our approach, however, offers no insight into the possible mechanisms that led to VOC emergence, and this clearly represents a limitation. Another shortcoming of this study is that, either for SARS-CoV-2 or for other viruses, most analyses relied on the comparison with a genome that is taken as the reference (the earliest sampled complete genome) but does not necessarily represent the ancestor of all circulating strains. Also, the nonindependence among viral genomes is only partially accounted for. Nonetheless, our findings add information to the existing knowledge of SARS-CoV-2 genetic diversity and help understand its evolution since entering human populations. Specifically, we suggest that the substitution spectrum of the virus is determined by an interplay of factors, including intrinsic biases in the replication process, avoidance of CpG dinucleotides and possibly other constraints exerted by the new host. These observations, if followed up by experimental analysis, can contribute to elucidate the selection pressures that are shaping the virus population.

## Data availability

The data that support the findings of this study are available from the GISAID and the NCBI databases. NCBI sequence IDs are listed in Supplementary Table 1.

**Key Points**

- Most coronaviruses display a mutation spectrum dominated by C to U and G to U substitutions—a feature that is not shared by other positive-sense RNA viruses.
- Analysis of the sequence context of C to U substitutions showed little evidence of APOBEC-mediated editing.
- C to U and G to U changes affect CpG dinucleotides at a frequency higher than expected.
- During the first months of SARS-CoV-2 pandemic spread, the frequency of both G to U and C to U substitutions increased.

## Supplementary Material

supplementary_figures_bbab382

Supplementary_table_1_bbab382

## Notes

**Diego Forni** is a researcher at IRCCS E. Medea. He is interested in the evolutionary history of human viruses and in the conflict between hosts and pathogens.

**Rachele Cagliani** is a researcher at IRCCS E. Medea, Italy. Her research mainly focuses on evolutionary history of human viruses and host–pathogen evolutionary conflict.

**Chiara Pontremoli** is a post-doc researcher at IRCCS E. Medea. She is interested in human virus evolution and she has experience in molecular biology techniques.

**Mario Clerici** is a Professor of Immunology at the University of Milan. His research focuses on the immunopathogenesis and the genetic background of human diseases.

**Manuela Sironi** is a PI in the computational biology unit at IRCCS E.Medea. Her major research interests include the evolutionary dynamics of human viruses and genetic conflicts.
