# Supplementary material for: The substitution spectra of coronavirus genomes
Source: Brief Bioinform. 2021 Sep 13;23(1):bbab382. doi: 10.1093/bib/bbab382 (PMC8499949; doi:10.1093/bib/bbab382)

Supplementary Figure 1. SARS-CoV-2 substitution spectrum. Distribution of SARS-CoV-2 substitutions grouped by ORF types. All substitution frequencies are reported after normalization by base frequency and by overall number of mutations of the respective ORF type. Data are plotted as mean and standard deviation of 100 sets of 1,000 genomes each. Substitution counts were as follows (mean and standard deviation):
Orf1ab: AC=6.78±1.93, AG=68.39±6.06, AT=10.79±2.25, CA=8.782.03, CG=0.37±0.58, CT=458.92±14.59, GA=41.85±4.36, GC=2.94±0.96, GT=110.48+7.02, TA=6.83±2.07, TC=57.52+5.32, TG=5.23±1.42;
Structural proteins: AC=4.21±1.21, AG=14.13±2.52, AT=8.10±1.69, CA=11.56±2.12, CG=3.43±1.22, CT=147.39±8.26, GA=21.59±2.83, GC=16.44±2.35, GT=72.55±5.72, TA=3.19±1.15, TC=25.98±2.96, TG=2.90±0.81;
Accessory proteins: AC=1.55±0.72, AG=8.36±2.12, AT=4.34±1.31, CA=2.39±1.01, CG=0.23±0.44, CT=69.20±5.58, GA=4.97±1.70, GC=2.93±0.99, GT=45.73±3.92, TA=0.720.85, TC=13.09±2.72, TG=1.68±0077

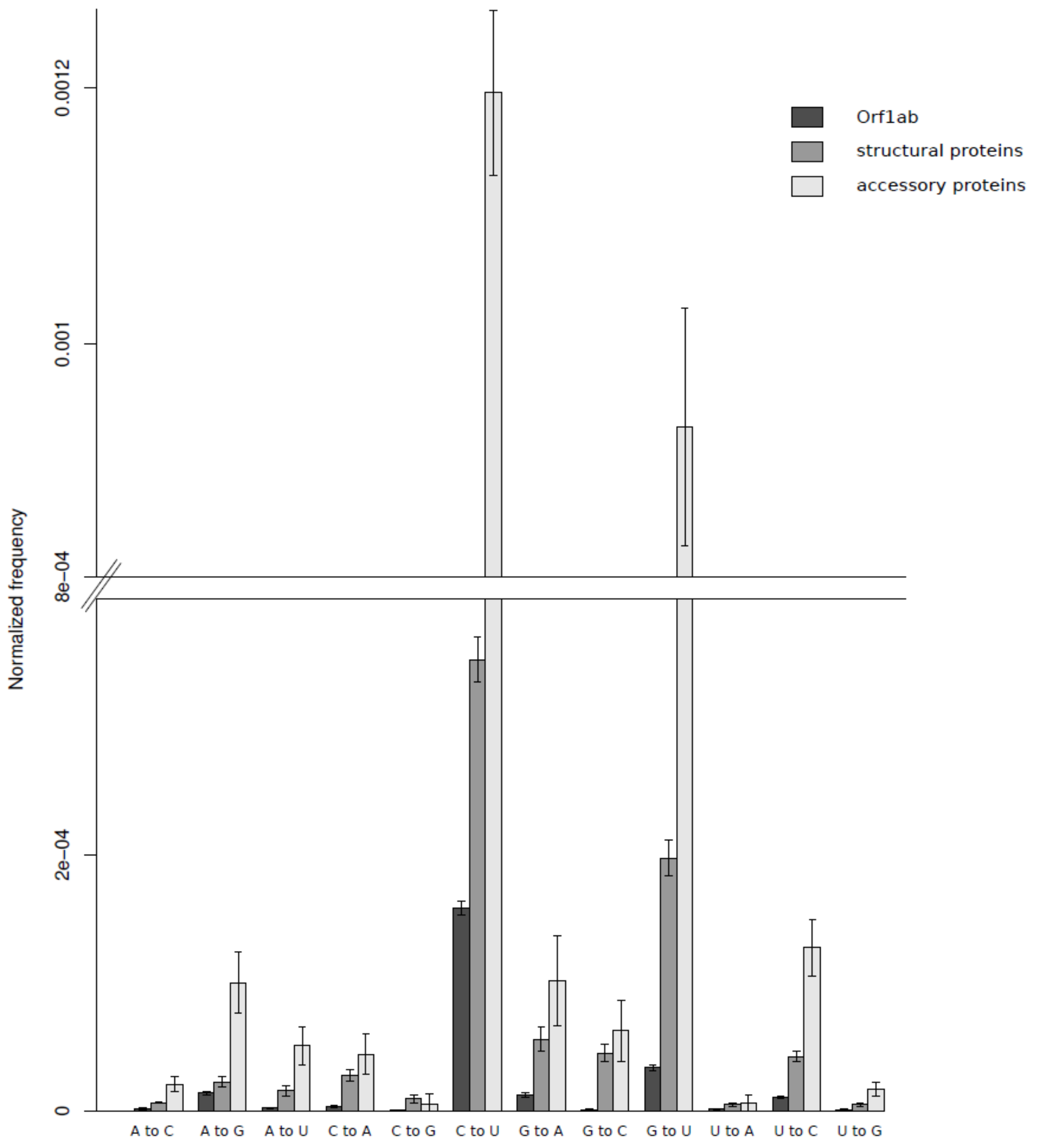

Supplementary Figure 2. C to U and G to U substitution frequencies for SARS-CoV-2 and SARS-CoV.

Comparison of the C to U and G to U frequencies in SARS-CoV (48 samples), and SARS-CoV-2 (10 sets of 48 random samples). Substitution frequencies are reported after normalization by base frequency and by overall number of mutations. Data for SARS-CoV-2 are plotted as mean and standard deviation.

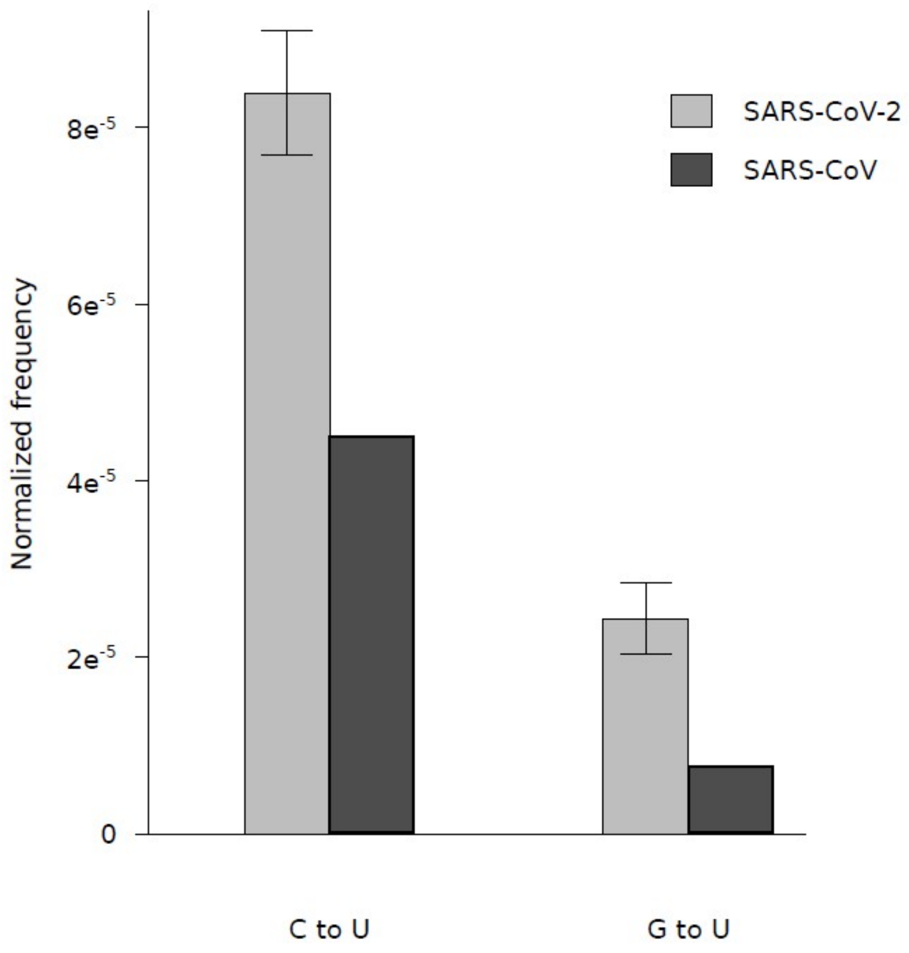

Supplementary Figure 3. Sequence context of C to U synonymous substitutions in SARS-CoV-2. Bars represent the frequency of substitutions that occur at fourfold degenerate NUC codons compared to fourfold degenerate NSC codons (where S is C or G). All data are plotted as mean and standard deviation of 100 sets of 1,000 genomes each.

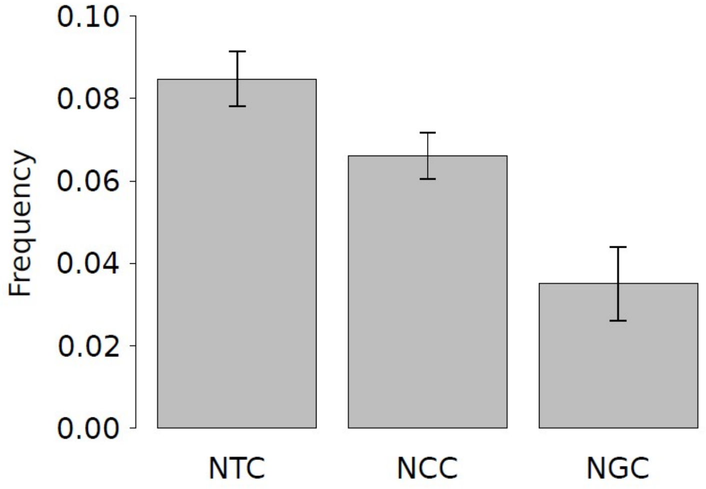

Supplementary Figure 4. Change of the substitution spectrum over time in RdRp mutants. Frequency of C to U and G to U mutations that have appeared per month in sequences carrying the P323L mutation in the viral RNA polymerase. Frequencies are normalized by the frequency of the changing nucleotide and by the overall number of changes occurring each month. Five hundred random sequences were used in each month. From July to November 2020 data are omitted to the low number of sequences/month in both groups.

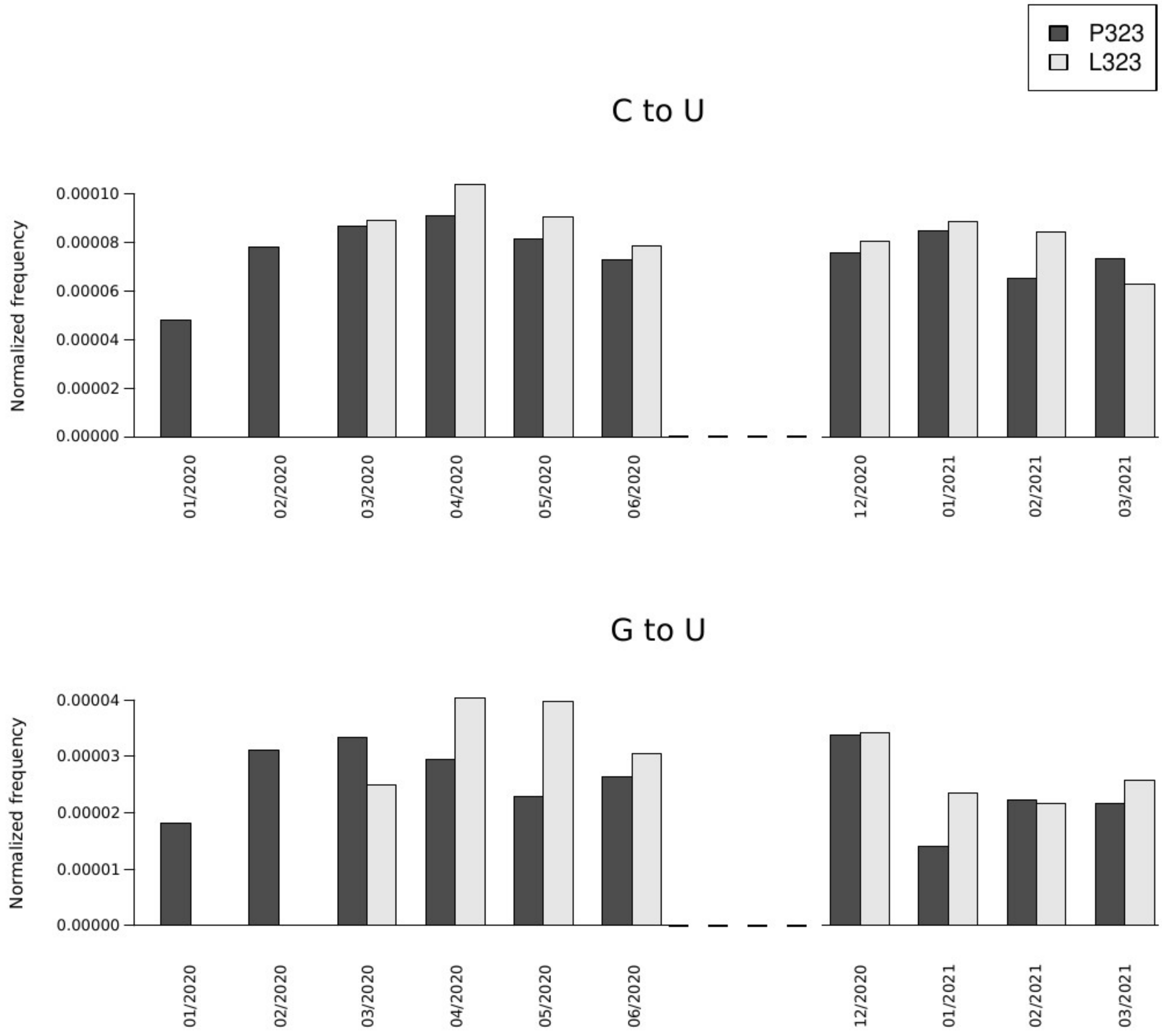

Supplement: supplementary_figures_bbab382 [file supplementary_figures_bbab382.pdf]
